# Reduced cortical thickness related to single nucleotide polymorphisms in the major histocompatibility complex region in antipsychotic‐naive schizophrenia

**DOI:** 10.1002/brb3.1253

**Published:** 2019-03-28

**Authors:** Bo Tao, Yuan Xiao, Na Hu, Chandan Shah, Lu Liu, Xin Gao, Jieke Liu, Wenjing Zhang, Li Yao, Heng Xu, Jun Hua, Su Lui

**Affiliations:** ^1^ Department of Radiology, Center for Medical Imaging West China Hospital of Sichuan University Chengdu China; ^2^ State Key Laboratory of Biotherapy Sichuan University Chengdu China; ^3^ Department of Radiology Johns Hopkins University of Medicine Baltimore Maryland

**Keywords:** antipsychotic‐naive schizophrenia, cortical thickness, magnetic resonance imaging, major histocompatibility complex, single nucleotide polymorphisms

## Abstract

**Instructions**

The aim of this study was to explore the relationships between changes in cortical thickness and single nucleotide polymorphisms (SNPs) in the major histocompatibility complex (MHC) region in a group of antipsychotic‐naive schizophrenia (AN‐SCZ) patients.

**Methods:**

Twenty‐five AN‐SCZ patients and 51 healthy controls (HCs) participated in this study. General linear models were used to identify associations between the average cortical thicknesses of each brain region (*N* = 68) and each of the 11 SNPs in the MHC region in the AN‐SCZ patients and HCs. Next, we performed independent‐sample *t* tests to investigate whether cortical thickness was significantly lower in the AN‐SCZ patients than in HCs in the brain regions that were significantly associated with the SNPs. Finally, we examined the correlations between clinical symptoms and cortical thickness in the above brain areas in the whole AN‐SCZ group using Pearson correlation tests.

**Results:**

Seven of the 11 SNPs within the MHC region were significantly associated with cortical thickness only in the AN‐SCZ patients; these included rs1635, rs1736913, rs2021722, rs204999, rs2523722, rs3131296, and rs9272105. The AN‐SCZ patients had significantly thinner cortical thicknesses in the above brain regions, especially the prefrontal cortex. Furthermore, the left entorhinal region was negatively correlated with Positive and Negative Symptom Scale (PANSS) activation scores in the AN‐SCZ group (*r* = −0.601, *p* = 0.03).

**Conclusions:**

This study provides evidence demonstrating the potential effects of MHC risk variants in cortical thickness deficits in AN‐SCZ. These data also support the notion that the immune system plays critical roles in the pathology of schizophrenia, which is mediated via the modulation of the development of cerebral cortical structures.

## 1 INTRODUCTION

The theory that immune dysregulation is a key factor in the pathogenesis of schizophrenia has been widely accepted. Evidence from epidemiology and postmortem and animal model studies has suggested that common variants in immune‐related genes and/or the altered expression of immune‐related genes and immunologic responses may be associated with abnormal neurodevelopment processes that begin long before the onset of the clinical presentation of schizophrenia (Alan & Elena, 2010; Fatemi & Folsom, 2009). Moreover, increasing numbers of genome‐wide association studies have indicated that single nucleotide polymorphisms (SNPs) in the major histocompatibility complex (MHC) region are significantly associated with schizophrenia (Purcell et al., 2009; Shi et al., 2009; Stefansson et al., 2009).

In addition to its vital role in immune functions, the *MHC* gene family is also important in the promotion of regular brain development and brain functional maturation (Corriveau, Huh, & Shatz, 1998; Huh et al., 2000). It has been reported that the dysregulation of these molecules may be associated with abnormal neurodevelopment in schizophrenia (Kinberley, 2014) as well as widespread brain deficits in regions including the prefrontal and temporal regions (Debnath, Cannon, & Venkatasubramanian, 2013; Shatz, 2009). These findings have been supported by studies performed using mouse models of *MHC Class I *deficiencies (Huh et al., 2000) and studies of schizophrenia patients (Agartz et al., 2011), both of which exhibited enlargement of the ventricles. However, it remains unclear whether alterations in *MHC *molecules could be related to the regional abnormalities in the gray matter observed in schizophrenia.

Imaging genetics has been successfully applied to the exploration of the associations between brain structural abnormalities and schizophrenia susceptibility genes in recent decades. This method usually requires much smaller sample sizes than are required by clinical studies, for which sample sizes of 20–100 patients are considered sufficient to obtain consistent findings (Hariri & Weinberger, 2003). However, few imaging genetics studies have directly explored the influence of *MHC* variations in brain anatomy in schizophrenia. One study conducted by Angartz et al. found that SNPs in rs2596532 in the MHC region were significantly associated with cerebral ventricular size in schizophrenia patients (Angartz et al., 2011). Two studies performed using the region of interest method showed that common variants in the MHC gene family had a significant relationship with decreased thalamus and hippocampal volumes in schizophrenia patients (Brucato, Guadalupe, Franke, Fisher, & Francks, 2015; Walters et al., 2013). In addition, the schizophrenia patients in these studies mostly received antipsychotic treatment, and this might have had an impact on illness‐related gene expression, protein phosphorylation, and new protein synthesis in addition to further effects on the mediation cerebral development and functional maturation (Kari, Silje, Christine, & Vidar, 2017; Kim, Giusti‐Rodriguez, & Crowley, 2018).

In this study, we selected cortical thickness as the primary intermediate phenotype for the following reasons: (a) many neuroimaging studies have detected cortical atrophy in the whole brains of schizophrenia patients, primarily in the frontal, temporal, and parietal regions (Goldman et al., 2009); (b) cortical thickness is a reliable indicator with high heritability, as demonstrated by many sibling and family MRI studies (Gogtay et al., 2007; Goldman et al., 2009; Winkler et al., 2010); and (c) compared with gray matter volume, cortical thickness may be more applicable to genetic imaging studies (Winkler et al., 2010).

Thus, we aimed to directly explore the relationship between changes in cortical thickness and SNPs of the MHC region at the whole‐brain level in schizophrenia patients. Furthermore, we enrolled a group of antipsychotic‐naive schizophrenia (AN‐SCZ) patients in the study to remove any potential interference caused by antipsychotics.

## 2 METHODS

### 2.1 Participants

This study was approved by the ethics committee of the West China Hospital of Sichuan University. All participants provided informed consent before participation. Twenty‐five chronically ill AN‐SCZ patients and 51 HCs were recruited and studied from May 2014 to July 2016. All participants were of Han ancestry and right‐handed. Clinical diagnoses of AN‐SCZ were made by an experienced psychiatrist on our research team using the Structured Clinical Interview for DSM‐IV Axis I Disorders (SCID). The Positive and Negative Symptom Scale (PANSS) was used to evaluate the severity of psychiatric symptoms in the AN‐SCZ patients before scanning (Kay, Fiszbein, & Opler, 1987). In addition, all AN‐SCZ patients met the following inclusion criteria: (a) aged from 18 to 60 years old, (b) no history of substance abuse or dependence, (c) no history of head injury or other neurologic or systemic illness, (d) no contraindications to MR scanning, and (e) an IQ score of at least 70.

These never‐treated patients were mostly identified from the Mental Health Screening Program of West China Hospital of Sichuan University. This project was designed to provide psychiatric care to individuals with serious mental illness who have never accepted antipsychotic treatment. Fifteen patients were recruited from rural regions in Chengdu City of West China, and the others lived in urban or suburban areas. On the basis of the available retrospective information, which was supported by family members and medical records, these patients were considered to have not received any treatment with psychiatric medications for the following reasons: parental concern about family stigma, a lack of understanding of the severity of the patient’s mental illness, poor socioeconomic conditions that limited travel and funds for medical care, and rejection of antipsychotics treatment when the patient was brought to medical attention for the first time near the time of illness onset.

The members of the comparison population were recruited from the same community or region as the patients by poster advertisements. The nonpatient edition of the SCID was used to ensure a lifetime absence of mental illness, and individuals with a history of major psychiatric illness in their first‐degree relatives were also excluded. Detailed information about the AN‐SCZ patients and comparison subjects, including age, sex, education, and the duration of illness, is shown in Table 1.

**Table 1:** Demographic and clinical characteristic of the AN‐SCZs and HCs

	AN‐SCZs (*N* = 25)	HCs (*N* = 51)	*p*‐Value
Age	49.64 ± 12.32	44.88 ± 5.32	0.075
Gender (M/F)	11/14	27/24	0.366
Education (years)	7.80 ± 1.69	8.90 ± 2.62	0.058
Duration (years)	21.60 ± 12.87		
PANSS total	91.45 ± 10.81		
Positive symptoms	24.83 ± 5.37		
Negative symptoms	23.08 ± 5.89		
General psychopathology symptoms	43.33 ± 5.79		
Thought disturbance	13.87 ± 2.91		
Activation	9.25 ± 1.72		
Paranoid	9.79 ± 3.19		
Depression	6.70 ± 2.74		
Anergia	10.46 ± 3.53		
Impulsive aggression	8.25 ± 2.57		

AN‐SCZs: antipsychotic‐naive schizophrenia patients; HCs: healthy controls; PANSS: positive and negative symptom scale.

### 2.2 Genotyping

Initially, we selected a group of SNPs located in the MHC region that exhibited significant associations with schizophrenia in the combined studies of the International Schizophrenia Consortium and Molecular Genetics of Schizophrenia (Corvin & Morris, 2014; Ripke et al., 2013; Shi et al., 2009). These SNPs included rs1635, rs20499, rs886424, rs926300, rs1736913, rs2021722, rs2523722, rs3131296, rs3800307, rs3800316, rs6904071, rs6913660, rs6923590, rs1321918, rs9272105, rs7746922, rs1319405, and rs9277219. Next, we excluded seven of the above SNPs based on the standard of the NCBI (http//www.ncbi.nlm.nih.gov/), which states that the minor allele frequency of each SNP must be at least 5%. Then, panels of 11, including rs1635, rs1736913, rs204999, rs9272105, rs886424, rs926300, rs1736913, rs2523722, rs3131296, rs3800316, and rs69322590, SNPs were presented. These SNPs were not in linkage disequilibrium (LD). Finally, genomic DNA was extracted from 5‐ml peripheral venous blood samples using a DNA extraction kit (TianGen Biotech Company, Beijing, China), and SNP genotyping was performed using the Sanger sequencing method.

### 2.3 MRI Acquisition and data processing

MRI data were obtained on a 3‐T GE Signa EXCITE scanner (General Electric, Milwaukee, WI) with an eight‐channel phase array head coil. High‐resolution T1‐weighted structural images were acquired with a volumetric three‐dimensional spoiled gradient recall sequence (repetition time = 8.5 ms, echo time = 3.4 ms, flip angle = 12°, field of view = 24 × 24 cm^2^, matrix = 256 × 256 × 128, and slice thickness = 1 mm).

The atlas‐based FreeSurfer software suite (http://surfer.nmr.mgh.harvard.edu, version 5.3) was applied for cortical modeling and volumetric segmentation of these structural MRI data. Cortical surface reconstructions were performed via an orderly process that included automated Talairach transformation, segmentation of the subcortical white matter and deep gray matter volumetric structures, intensity normalization, tessellation of the gray matter and white matter boundaries, and automated topology correction and surface deformation following intensity gradients to optimally place the gray/white and gray/CSF borders at the locations with the greatest defined shifts in intensity (Fischl & Dale, 2000). Once the cortical maps were finished, cortical thicknesses were calculated as the closest distance from the gray/white boundary to the gray/CSF boundary at each vertex on the tessellated surface (Fischl & Dale, 2000).

### 2.4 Statistical analysis

In the graphical interface of FreeSurfer (termed Query, Design, Estimate, Contrast), a Gaussian kernel with a full‐width at half‐maximum of 10 mm was applied during the smoothing procedure to the cortical thickness data obtained from all subjects. These were averaged to the common spherical coordinate system. Next, we extracted and averaged the values of the cortical thickness of each brain region (*N* = 68) for each participant. Then, general linear models were used to explore the relationship between each of the above‐mentioned SNPs with cortical thickness values in the AN‐SCZ and HC groups, with age and gender incorporated in the models as covariates (*y* = β_1_X_1_ + β_2_X_2_ + β_3_X_3_ + β_0_，*y*: cortical thickness，X_1_: SNP, X_2_: age, X_3_: gender). The statistical results were corrected for multiple comparisons using the false‐discovery rate with a threshold of 0.05 (11 SNPs × 68 brain regions × 2 groups). The average cortical thicknesses of all cerebral regions in the AN‐SCZs and HCs were normally distributed (*Z* = 0.451 and 0.783, respectively; *p* > 0.05). In the next step, we applied an independent‐samples *t test* with a threshold of 0.05 to explore whether cortical thicknesses differed significantly between the AN‐SCZs and HCs in the brain regions that were significantly associated with the SNPs of the MHC region. Finally, we examined the correlations between clinical symptoms and cortical thickness in the above brain areas in the whole AN‐SCZ group using Pearson correlation tests.

## 3 RESULTS

### 3.1 SNPs in the MHC region and cortical thickness were significantly associated

In the absence of the influence of antipsychotics, the general linear models revealed that there was a strong association between SNPs in the MHC region and cortical thickness in the AN‐SCZs but not in the HCs. These SNPs included rs1635, rs1736913, rs2021722, rs204999, rs2523722, rs3131296, and rs9272105. The SNP rs1635 was significantly associated with cortical thickness in the area surrounding the calcarine sulcus (*p* = 0.02). The SNP rs1736913 was related to the right insular (*p* = 0.03) and the left superior temporal gyri (*p* = 0.03) in the AN‐SCZs, and rs2021722 was strongly correlated with the cortical thicknesses of the left parahippocampal gyrus (*p* = 0.04) and the left entorhinal cortex (*p* = 0.03). Moreover, the models revealed that rs3131296 was related to cortical thickness in the pars orbitalis part of the left inferior frontal cortex (*p* = 0.01) and the right medial orbitofrontal cortex (*p* = 0.05). However, rs204999 was only associated with cortical thickness in the caudal part of the anterior cingulate cortex (*p* = 0.04). Among these seven SNPs in the MHC region, rs9272105 produced the most remarkable findings. This SNP was significantly associated with cortical gray matter thickness in up to 10 brain areas, including the right frontal pole (*p* = 0.001), the right rostral middle frontal cortex (*p* = 0.005), the right pars triangularis (*p* = 0.02), the right pars orbitalis (*p* = 0.02), and other areas, as described in detail in Table 2. Regression models showed that there was no significant association between SNPs of the MHC region and cortical thickness in the HCs.

**Table 2:** Significant associations between SNPs within the MHC region and cortical thickness in AN‐SCZs and HCs

SNP	Region	AN‐SCZs (*N* = 25)		HCs (*N* = 51)	
		*p*‐Value	Coefficient	*p*‐Value	Coefficient
rs1635	rh_pericalcarine	0.021	−0.079	0.194	−0.042
rs1736913	rh_insula	0.032	−0.139	0.286	0.034
rs1736913	lh_superior temporal	0.034	0.119	0.468	−0.020
rs2021722	lh_entorhinal	0.030	0.39	0.253	0.316
rs2021722	lh_parahippocampal	0.037	0.253	0.211	−0.225
rs204999	rh_caudal anterior cingulate	0.037	0.445	0.802	−0.018
rs2523722	lh_isthmus cingulate	0.009	0.255	0.734	0.044
rs3131296	lh_pars orbitalis	0.010	0.641	0.569	−0.164
rs3131296	rh_medial orbitofrontal	0.048	0.347	0.802	−0.054
rs9272105	rh_frontal pole	0.001	0.922	0.225	0.227
rs9272105	rh_rostral middle frontal	0.005	0.289	0.750	0.021
rs9272105	lh_transverse temporal	0.028	0.313	0.839	−0.024
rs9272105	rh_lateral orbitofrontal	0.030	0.302	0.307	0.112
rs9272105	rh_cuneus	0.033	0.18	0.245	0.089
rs9272105	rh_medial orbitofrontal	0.041	0.254	0.640	0.059
rs9272105	rh_lateral occipital	0.047	0.153	0.226	0.102
rs9272105	lh_rostral middle frontal	0.013	0.229	0.964	0.003
rs9272105	lh_pars triangularis	0.019	0.226	0.535	0.065
rs9272105	rh_pars orbitalis	0.020	0.269	0.468	0.041

AN‐SCZs: antipsychotic‐naive schizophrenia patients; HCs: healthy controls; SNPs: single nucleotide polymorphisms; MHC: major histocompatibility complex.

### 3.2 Significant differences in cortical thickness in “relevant association” brain regions

Independent‐sample *t tests *of the differences in cortical thickness between the AN‐SCZs and HCs showed that nine of the 18 cerebral areas that were strongly associated with the SNPs evaluated in this study exhibited significant differences between the two groups (*p* < 0.05). In other words, the AN‐SCZs exhibited reduced cortical thicknesses in nine brain regions, including the left entorhinal cortex, the left pars triangularis cortex, the left rostral middle frontal cortex, the right lateral occipital cortex, the right medial orbitofrontal cortex, the gray matter surrounding the right calcarine gyrus, the right rostral middle frontal cortex, the right frontal pole, and the caudal part of the right anterior cingulate cortex (Table 3). Moreover, the right transverse temporal region and the right pars orbital cortex exhibited trends toward decreased cortical thicknesses in the AN‐SCZs (*p* = 0.053 and 0.051, respectively).

**Table 3:** Significant differences in cortical thickness in the above brain regions between the AN‐SCZs and HCs

Brain region	AN‐SCZs (*N* = 25)	HCs (*N* = 51)	T	*p*‐Value
	Thickness (mean ± *SD*)	Thickness (mean ± *SD*)		
lh_entorhinal	2.739470 ± 0.393674	2.950720 ± 0.395327	2.192	0.033
lh_rostral middle frontal	2.21640 ± 0.128098	2.33137 ± 0.136965	−3.51	0.001
lh_pars triangularis	2.379710 ± 0.180205	2.250080 ± 0.138514	−3.459	0.002
rh_pericalcarine	1.661 ± 0.123734	1.73671 ± 0.148349	−2.202	0.031
rh_caudal anterior cingulate	1.99661 ± 0.203454	2.18096 ± 0.278000	3.279	0.002
rh_frontal pole	2.59124 ± 0.419035	2.80055 ± 0.331422	−2.367	0.021
rh_rostral middle frontal	2.21544 ± 0.143016	2.34447 ± 0.119940	−4.133	0.000
rh_medial orbitofrontal	2.3186 ± 0.176709	2.41671 ± 0.210023	−2.011	0.048
rh_lateral occipital	2.13512 ± 0.100834	2.23212 ± 0.139072	−3.106	0.003

AN‐SCZs: antipsychotic‐naive schizophrenia patients; HCs: healthy controls.

### 3.3 Correlations between clinical symptoms and reduced cortical thickness in “relevant association” brain regions

The thickness of the left entorhinal region was negatively correlated with PANSS activation scores in the AN‐SCZs (r = −0.601, *p* = 0.03).

## 4 DISCUSSION

This study explores the significant relationships between cortical thickness and SNPs in the MHC gene family in a group of AN‐SCZ patients; our findings support a link between SNPs in the MHC region and cortical thickness in the frontal and temporal regions. Furthermore, the thicknesses of these regions were lower in the AN‐SCZs than in the HCs. These data also show that in the AN‐SCZs, cerebral deficits in the left entorhinal region were also relevant to activation. Moreover, these results are in accordance with previous findings that demonstrated that genetic variation within the MHC region is implicated in the characteristic brain abnormalities observed in schizophrenia (Agartz et al., 2011; Brucato et al., 2015; Walters et al., 2013).

The most interesting finding in this study is that seven SNPs of the MHC region, including rs1635, rs1736913, rs2021722, rs204999, rs2523722, rs3131296 and rs9272105, were related to decreased cortical thicknesses, especially in the prefrontal cortex (PFC), in the AN‐SCZs. Given the complex LD structure of the MHC region, it remains extremely challenging to identify the direct pathophysiological mechanism by which these genetic variants influence cerebral anatomy in schizophrenia. However, previous studies have shown that molecular variations in the MHC genes may alter the development of neurons and their neurites (i.e., axons and dendrites), mainly affecting cortical thickness. For example, rs3131296 is located in the largest intron (intron 18) of the neurogenic locus notch homolog protein 4 (*NOTCH4*), which is one of the top candidate genes for schizophrenia (Allen et al., 2008). This SNP can affect the efficiency and/or alternative splicing of transcripts of *NOTCH1* and *NOTCH2 *(Shayevitz, Cohen, Stephen, & Glatt, 2012; Thomas, Sikich, Lieberman, & LaMantia, 2001), and the *NOTCH1* and *NOTCH2 *signaling pathways are known to be associated with cortical neurite growth via their roles in mediating the numbers of interneuronal contacts (Sestan, Tsakonas, & Rakic, 1999). Another SNP, rs1635, is located in exon 1 of *NFKB*‐activating protein‐like (*NKAPL*) and encodes a threonine‐to‐glutamine substitution; this mutation may cause abnormal functioning of the *NKAPL *protein (Wu et al., 2017). Moreover, studies have suggested that *NKAPL *may control specific aspects of neurogenesis and may affect cortical growth by mediating the *Notch *and *NF‐kB* signaling pathways (Luke & Kaltschmidtib, 1997; Shayevitz et al., 2012; Worlitzer & Schwamborn, 2014). Hence, polymorphisms of rs1635 could alter the function of the *NKAPL* protein and thereby influence cortical development. The third SNP, rs9272105, is located approximately 5 kbs upstream of the coding gene *HLA‐DQA1*, which is one of the most common cell adhesion molecule (CAM)‐related genes (Hargreaves et al., 2014). This SNP may alter early human cortical development by up‐ or downregulating neutral‐CAM (*NCAM*) (Cox et al., 2009). Moreover, schizophrenia and other neuropsychiatric disorders are associated with the dysregulation of *NCAM* in the hippocampus, cortex, and subcortical structures (Marquis, 2000). Regarding the other four SNPs, that is, rs1736913, rs204999, rs2021722, and rs2523722, they also exhibited significant relationships with schizophrenia in this study, consistent with the results of previous studies (Corvin & Morris, 2014; Irish Schizophrenia Genomics Consortium, 2012; Saito et al., 2014). Our findings extend those of previous studies by directly supporting the associations between MHC variants and cortical thickness in schizophrenia patients in vivo.

Among the 16 regions that displayed associations with SNP variations within the MHC region, the cortical thicknesses of nine were significantly thinner in the AN‐SCZs, and these areas were mainly centralized in the PFC. These findings revealing reduced cortical thicknesses in the PFC are consistent with those of previous studies (Kuperberg, 2003; Schultz et al., 2010; Venkatasubramanian, Koch, Wagner, & Keshavan, 2008). The PFC is known to send and receive connections from nearly all cortical and subcortical structures, especially the limbic system. The PFC is critical for the top‐down control of behavior and responsible for executive function, working memory and emotional evaluation (Earl & Cohen, 2001), as supported by many studies that have demonstrated that the cognitive impairments observed in schizophrenia are associated with PFC dysfunction (Callicott et al., 2003; Deamma et al., 2001). Furthermore, a reduction in the cortical thickness of the left entorhinal region was negatively correlated with the severity of activation in our study. It has been suggested that the entorhinal cortex is crucial for memory processing, associative learning, and spatial navigation (Charles, Browning, & Gaffan, 2004; Hafting, Fyhn, Molden, Moser, & Moser, 2005). Moreover, many studies have reported that smaller left entorhinal cortex volumes are associated with clinical symptoms, especially delusions (Baiano et al., 2008; Prasad, Patel, Muddasani, Sweeney, & Keshavan, 2004).

Together, these data suggest that SNPs in the MHC region may alter brain anatomy and thereby affect clinical symptoms in schizophrenia. Thus, our study provides in vivo evidence supporting the theory that the immune system is involved in the pathogenesis of schizophrenia.

Two issues should be addressed when explaining the current findings. First, the sample size in our study was relatively small due to difficulties associated with simultaneously collecting imaging data and blood in antipsychotic‐naïve schizophrenia patients. Second, due to the high LD of the MHC region, its effects on brain structure and function are highly complex. Future studies should focus on more specific SNPs that exhibit significant associations with schizophrenia in the Chinese Han population. Moreover, a validation group of case‐control studies should be used to enhance the reliability of this research.

## 5 CONCLUSIONS

In the absence of the influence of antipsychotics, this study provides evidence demonstrating the effects of MHC risk variants in cortical thickness deficits in schizophrenia patients. This study also supports the notion that the immune system plays a critical role in the pathology of schizophrenia, which is mediated by the modulation of the development of cerebral cortical structures. Future longitudinal studies of much larger sample populations are encouraged to confirm our findings and further investigate the trajectory of the disease.

## CONFLICT OF INTEREST

The authors report that they have no conflicts of interest.

## AUTHORS CONTRIBUTIONS

Su Lui had the original idea for this project and the development of the study design. Li Yao and Wenjing Zhang conducted the search of previous papers. Lu Liu, Xin Gao, Jieke Liu, Chandan Shah, and Qiyong Gong collected and analyzed the data. Bo Tao and Yuan Xiao drafted the manuscript, which was revised by all authors. Heng Xu and Jun Hua helped to interpret the results and critically revised the manuscript.
